# Short-chain fatty acids suppresses astrocyte activation by amplifying Trp-AhR-AQP4 signaling in experimental autoimmune encephalomyelitis mice

**DOI:** 10.1007/s00018-024-05332-x

**Published:** 2024-07-08

**Authors:** Xiuli Lin, Yufeng Peng, Zhimei Guo, Wuhui He, Wenyuan Guo, Junmin Feng, Lin Lu, Qin Liu, Pingyi Xu

**Affiliations:** 1 Department of Neurology, The First Affiliated Hospital of Guangzhou Medical University, Guangzhou, 510000 Guangdong China; 2 Department of Neurology, The Seventh Affiliated Hospital of Sun Yat-sen University, Shenzhen, Guangdong China; 3 Department of Otolaryngology, Sun Yat-sen Memorial Hospital, Sun Yat-sen University, Guangzhou, Guangdong China

**Keywords:** Multiple sclerosis, Short-chain fatty acids, Astrocytes, Trp-AhR-AQP4 signaling, BBB-glymphatic system

## Abstract

The function of astrocytes in response to gut microbiota-derived signals has an important role in the pathophysiological processes of central nervous system (CNS) diseases. However, the specific effects of microbiota-derived metabolites on astrocyte activation have not been elucidated yet. Experimental autoimmune encephalomyelitis (EAE) was induced in female C57BL/6 mice as a classical MS model. The alterations of gut microbiota and the levels of short-chain fatty acids (SCFAs) were assessed after EAE induction. We observed that EAE mice exhibit low levels of *Allobaculum*, *Clostridium_IV*, *Clostridium_XlVb*, *Lactobacillus* genera, and microbial-derived SCFAs metabolites. SCFAs supplementation suppressed astrocyte activation by increasing the level of tryptophan (Trp)-derived AhR ligands that activating the AhR. The beneficial effects of SCFAs supplementation on the clinical scores, histopathological alterations, and the blood brain barrier (BBB)-glymphatic function were abolished by intracisterna magna injection of AAV-GFAP-shAhR. Moreover, SCFAs supplementation suppressed the loss of AQP4 polarity within astrocytes in an AhR-dependent manner. Together, SCFAs potentially suppresses astrocyte activation by amplifying Trp-AhR-AQP4 signaling in EAE mice. Our study demonstrates that SCFAs supplementation may serve as a viable therapy for inflammatory disorders of the CNS.

**Supplementary Information:**

The online version contains supplementary material available at 10.1007/s00018-024-05332-x.

## Introduction

The microbiota–gut–brain communication refers to the bidirectional signaling mechanisms between the gastrointestinal tract and the central nervous system (CNS), highlighting the key role of gut microbiota-derived signals in the pathophysiological processes in the CNS [1, 2]. Short-chain fatty acids (SCFAs) are among the most well-characterized microbiota-derived metabolites, serving as potent immune-modulatory compounds that modulate the inflammatory responses of lymphocytes, neutrophils, and macrophage in both the periphery and the CNS [3, 4]. The alterations in SCFAs have been implicated in the pathogenesis of various CNS diseases, including stroke, traumatic brain injury and Parkinson disease via microbiota–gut–brain communication [5–7]. Multiple sclerosis (MS) is a chronic inflammatory, demyelinating autoimmune disease in the CNS [8]. Our recent research has demonstrated that SCFAs-producing microbiota intervention reduced the Th17 response and increased the Treg response in the CNS of experimental autoimmune encephalomyelitis (EAE) mice, a classical model of MS. These findings underscore the critical role of SCFAs in modulating neuroinflammatory diseases [9]. However, in comparison to peripheral cells, the current understanding of how SCFAs regulate CNS cells in MS is relatively limited.

Astrocytes, abundantly residing in the CNS, play a pivotal role in the microbiota–gut–brain communication [10]. A recent study has indicated that gut microbiota dysbiosis triggers astrocyte dysfunction by suppressing the expression of circHIPK2, ultimately exacerbating depressive behavior in mice [11]. Meanwhile, astrocytes are crucial in the development of the blood–brain barrier (BBB) and glymphatic system, owing to their endfeet that tightly envelope cerebral microvessels [12, 13]. The BBB serves as a protective barrier that prevents toxins and pathogens from freely infiltrating the brain through the utilization of transport proteins that remove these substances from the brain and transport them back into the bloodstream. Simultaneously, the glymphatic system can efficiently clear waste from the interstitial space of the brain. Together, these mechanisms work synergistically to safeguard the intracerebral microenvironment, minimizing immune disturbance in MS [14, 15]. Upon the activation of astrocyte that leads to the BBB-glymphatic dysfunction, immune cells, antigens and pro-inflammatory cytokines might abnormally accumulate in the CNS and exacerbate immune damage in MS [13]. Recently, we have shown that the decreased abundance of *Lactobacillus* induced by constipation exacerbated BBB damage and EAE severity [16]. However, whether the activation of astrocyte is involved in the regulation of SCFAs in BBB-glymphatic system remains unknown.

The aryl-hydrocarbon receptor (AhR), expressed ubiquitously in astrocytes, participates in critical transcriptional processes within cells by binding gut microbiota-derived endogenous ligands [17, 18]. Tryptophan (Trp) can be metabolized into various AhR ligands, such as 5-hydroxyindoleacetate (5-HIAA), xanthurenic acid and riboflavin, and these processes are influenced by the intestinal microenvironment [19]. Recent evidence have reported that SCFA butyrate increased the level of 5-HIAA and mitigated the severity of rheumatoid arthritis [20]. Furthermore, researches have revealed a decrease in the levels of AhR endogenous agonists in patients with MS [21, 22]. These findings point to a potential involvement of AhR signaling in the pathogenesis of MS. Therefore, it is of significant interest to investigate whether SCFAs-induced Trp-AhR signaling exerts an influence on the activation of astrocyte in MS.

The water channel protein aquaporin-4 (AQP4), which is highly localized in the endfeet of astrocytes, is known as AQP4 polarity, which plays an important role in the differentiation and activation of astrocyte [23]. Additionally, polarized AQP4 in astrocytes facilitates paravascular cerebrospinal fluid (CSF)-interstitial fluid (ISF) exchange, contributing to the regulation of BBB-glymphatic function [24, 25]. Recent literature has reported that the loss of AQP4 polarity induces astrocyte activation, aggravating BBB damage and cerebral edema [26]. However, it remains unclear whether Trp-AhR signaling mediates astrocyte activation via AQP4.

In this study, we revealed that the levels of SCFAs-producing microbiota and their derived SCFAs were reduced in EAE mice. Supplementation with SCFAs enhanced the availability of Trp-derived AhR ligands that activated AhR and consequently suppressed the loss of AQP4 polarity and astrocyte activation. This ultimately led to an amelioration of BBB-glymphatic dysfunction and a reduction in EAE severity.

## Materials and methods

### Animals

Female C57BL/6 mice were obtained from the Experimental Animal Center of Guangdong (Guangzhou, China) and were kept in a pathogen-free facility at the Guangzhou Medical University. All experiments were approved by the Bioethics Committee of Guangzhou Medical University.

### EAE induction and SCFAs treatments

EAE was induced in 6–8-week-old female C57BL/6 mice [27] by subcutaneous immunization with 200 µg myelin oligodendrocyte glycoprotein (MOG_35–55_, GL Biochem Ltd, China) peptide emulsified in complete Freund’s adjuvant (CFA, Sigma-Aldrich, USA, F5881) including 500 μg Mycobacterium tuberculosis H37RA (BD Biosciences, USA, 231141) on day 0 and 7. Immediately thereafter and on day 2, the mice received an intraperitoneal injection of 300 ng pertussis toxin (PTX, KKL MED, USA, KM10754) in 100 μL PBS. Clinical signs of EAE were scored daily and blindly by two researchers individually ranging from 0 to 5 as follows: grade 5, death; grade 4.5, near death, moribund; grade 4, complete paralysis of two limbs; grade 3, complete paralysis of a single limb; grade 2.5, partial limb paralysis and ataxia; grade 2, dysfunctional gait with limp tail and ataxia; and grade 1, dysfunctional gait with tail tonicity or limp tail; 0, no signs of disease [16]. On day 0, the drinking water of mice was supplemented with SCFAs mix (sodium acetate 67.5 mM, 241245; sodium propionate 40 mM, P5436; and sodium butyrate 25.9 mM, 303410, Sigma–Aldrich, USA) and changed every 3 days as described [28]. A control group received only sodium chloride. Throughout the duration of the experiment, mice were continuously provided with SCFAs in their drinking water.

### 16S rRNA gene sequencing and bioinformatic analysis

Freshly extruded stools were collected immediately after the EAE induction for 16S rRNA sequencing analysis at BGI Co. (Shenzhen, China) following the methodology described in our previously published study [16]. Briefly, DNA was extracted using QuickGene DNA tissue kit from Kurabo Company (Neyagawa, Japan) and next used for PCR amplification and sequencing of the V3 and V4 region of bacterial 16S rRNA genes using Illumina MiSeq technology. Taxonomic annotation was performed using the GreenGene database. Partial least squares discrimination analysis (PLS-DA) was performed based on operational taxonomic units (OTU) abundance information using QIIME (Quantitative Insights into Microbial Ecology, version1.8.0). The differential microbial flora biomarkers among groups were performed using linear discriminant analysis (LDA) effect size (LEfSe) analysis.

### SCFAs quantification based on LC–MS

The freshly extruded stools and serum were collected on the 22nd day post-induction (p.i.) to initiate EAE. For fecal samples, 20 mg of the samples were accurately weighed and placed in EP tube. After adding 0.5% v/v phosphoric acid solution and a small steel ball, the mixture was ground, vortexed, and ultrasonicated. After centrifugation of the mixture at 12,000 rpm for 10 min at 4 °C, the supernatant was collected. 500 μL MTBE (containing internal standard) solution was added to the centrifugal tube.

For serum samples, the samples were thawed and vortexed for 1 min prior to analysis. Then, 50 μL of the sample was added to an eppendorf tube, followed by the addition of a 0.5% *v*/*v* phosphoric acid solution. The mixture was vortexed for 3 min. 150 μL MTBE (containing internal standard) solution was added.

Both the fecal and serum mixtures were vortexed and ultrasonicated separately. After ultrasonication, each mixture was centrifuged at 12,000 r/min for 10 min at 4 °C. The resulting supernatants obtained from the centrifugation of the fecal and serum samples were collected for SCFAs detection using the Agilent 7890B-7000D GC–MS/MS platform [29, 30].

### Large scale medical targeted metabolomics based on UHPLC-MS

The brain samples were collected on the 22nd day p.i. to initiate EAE. The metabolites were extracted from brain samples followed by incubation and centrifugation at 16,000 g and 4 °C for 20 min. The resulting supernatant was transferred to a sampling vial for subsequent analysis using UHPLC-MS. Quality control (QC) samples were prepared and analyzed using the same procedure as the experimental samples in each batch. The dried extracts were dissolved in 50% acetonitrile and stored at −80 °C until analysis.

UHPLC/MS was employed for metabolite detection in both electrospray negative-ionization and positive-ionization modes, utilizing a Shimadzu Nexera X2 LC-30AD system. The ACQUITY UPLC HSS T3 column (1.7 μm, 2.1 mm × 100 mm, Wasters) was used in this experiment. A gradient elution method was employed to separate the compounds. During the acquisition, QC samples were injected periodically to monitor the reproducibility of the analysis. Widely targeted metabolites were quantified using multiple-reaction monitoring mode set up as described in previous report [31, 32]. All transitions from large-scale metabolites were detected with optimized decluttering potential and collision energy. MultiQuant 3.0.2 software was used to extract the original MRM data of large-scale metabolites and obtain the peak area of each metabolite for quantification from different samples. The discriminating metabolites were obtained using a statistically significant threshold of variable influence on projection values obtained from the OPLS-DA model.

### RNA sequencing and bioinformatic analysis

For RNA from brain tissue, cDNA was generated using random oligonucleotides, Super Script II, DNA Polymerase I and RNase H. The library fragments were purified using the AMPure XP system (Beckman Coulter, Beverly, CA, USA). DNA fragments with ligated adaptor molecules on both ends were selectively enriched using Illumina PCR Primer Cocktail in a 15 cycle PCR reaction. Products were purified (AMPure XP system) and quantified using the Agilent high sensitivity DNA assay on a Bioanalyzer 2100 system (Agilent). The sequencing library was then sequenced on NovaSeq 6000 platform (Illumina) by Shanghai Personal Biotechnology Cp. Ltd.

### Quantitative real-time PCR

RNA from brain tissue was extracted using miRNeasy kits (Qiagen, Germany, #217,004). The extracted RNA was then reverse transcribed using the Evo M-MLV RT Master Mix kit (Accurate Biology, China, #AG11706). Subsequently, quantitative PCR (qPCR) was performed on the generated cDNA samples using the SYBR Green Pro Taq HS kit (Accurate Biology, China, #AG11701) and the real-time polymerase chain reaction system (Roche LightCycler 480). The primer sequences used for qPCR are listed in Table 1. β-actin was chosen as the internal control, and the comparative cycle threshold (ΔΔ*C*_t_) method was employed to determine gene expression levels.

**Table 1:** Primers used for RT-PCR

Target genes	Forward primers	Reverse primers	Source
	(5ʹ–3ʹ)	(5ʹ–3ʹ)	
*Gfap*	ACCAGCTTACGGCCAACAG	CCAGCGATTCAACCTTTCTCT	PrimerBank
*Serpina3n*	ATTTGTCCCAATGTCTGCGAA	TGGCTATCTTGGCTATAAAGGGG	
*S100b*	TGGTTGCCCTCATTGATGTCT	CCCATCCCCATCTTCGTCC	
*Mmp9*	GCAGAGGCATACTTGTACCG	TGATGTTATGATGGTCCCACTTG	

### Immunofluorescence

Brains were collected on the 22nd day p.i. to initiate EAE and were dissected and embedded into optimal cutting temperature compound (OCT, Tissue-Tek) and snap-frozen for cryo-sectioning. Slides were incubated in 1% Triton X-100 for 30 min and then incubated in 10% goat serum at room temperature for 30 min to block nonspecific antibody binding. For GFAP/AhR/AQP4 staining, the slices were incubated with primary antibodies against GFAP (1:1000, Cell Signaling Technology, USA, #3670), AhR (1:200, Boster Biological Technology, China, A00225-4) or AQP4 (1:1000, Proteintech, USA, CL488-16473) at 4 °C overnight after washing with PBS. Slices were then incubated with Alexa 488-conjugated (Cell Signaling Technology, USA, #4412S), Alexa 555-conjugated (Cell Signaling Technology, USA, #4409) secondary antibodies at a concentration of 1:1000 at room temperature for 2 h in the dark. The slides were mounted in Slowfade gold antifade mountant with DAPI (Thermo Fisher Scientific, USA, S36942). Slides were taken on an Leica DM6B microscope or Carl Zeiss 880 confocal microscope.

### Western blots analysis

Brain tissues isolated from mice were lysed with RIPA lysis buffer with 1% protease inhibitor cocktail, and supernatants were collected as total protein. Protein concentration was determined with the BCA Protein Assay Reagent. Lysates (100 μg) were separated by SDS-PAGE and transferred to PVDF membranes. After blocking with 5% BSA in TBS, membranes were incubated with the following primary antibodies: anti-AhR (1:500, Boster Biological Technology, China, A00225-4), anti-Claudin 5 (1:1000, Abcam, UK, ab131259), anti-Occludin (1:1000, Abcam, UK, ab167161), anti-ZO-1 (1:1000, Abcam, UK, ab276131), anti-AQP4 (1:1000, Proteintech, USA, 16473-1-AP), anti-GAPDH (1:5000, Affinity, USA, #AF7021), and anti-β-tubulin (1:5000, Affinity, USA, #AF7011). After incubation with a HRP-conjugated anti-rabbit secondary antibody (1:2000, Abcam, UK, ab97051), the proteins were visualized with ECL reagents (Millipore, USA, #WBKLS0100) for the immunoreactive bands visualization. The intensity of each target protein band was quantified by densitometry analysis using ImageJ software.

### Histopathology

Brains and lumbar spinal cords tissues were collected on the 22nd day p.i. to initiate EAE and were fixed by cardiac perfusion with 4% (w/v) PFA, and embedded in paraffin. Sections were stained with hematoxylin and eosin (HE) and luxol fast blue (LFB). ImageJ Software analysis (NIH, Bethesda, MD, USA) was used to evaluate inflammatory cell infiltration and demyelination.

### Flow cytometry

Brains and spinal cords tissues from different treated mice were collected on the 22nd day p.i. to initiate EAE for the flow cytometry analysis. Tissues were subjected to 0.5 mg/mL collagenase solution digestion at 37 °C for 30 min, then pressed through a 40 μm cell strainer. CNS-infiltrating mononuclear cells were separated from myelin debris by centrifugation in 30%/70% Percoll solution [16]. Collected cells were stained with CD4-BV510 (BD Biosciences, USA, 563106), IFN-γ-PE-Cy7 (BD Biosciences, USA, 557649) and IL-17A-BV421 (BD Biosciences, USA, 563354). Samples were measured by a fluorescence-activated cell sorter flow cytometer (BD Biosciences), and then analyzed by FlowJo (Tree Star, Ashland, OR).

### Transmission electron microscopy

Brains were isolated and fixed in electron microscope fixative (Servicebio) overnight at 4 °C for transmission electron microscopy (TEM) [16]. Specimens were then rinsed with phosphoric acid, dehydrated with acetone, immersed, embedded in epoxy resin, and cut into 60 nm ultrathin sections using an ultramicrotome (Leica, EM UC7). Grids were observed using a transmission electron microscope (FEI, Czech Republic).

### Evans blue perfusion

Mice were injected with 0.4% evans blue dye at a dose of 200 mg/kg into the tail vein and circulated for 30 min [16]. Then mice were fixed by cardiac perfusion with PBS and 4% (w/v) PFA. Brains were obtained and tissue cryo-sections were analyzed by fluorescence microscopy.

### Virus preparation

AAV8-GFAP-shAhR were purchased from (Sunbio Medical Biotechnology Company, China) at the titer of 1.0*10^13^ vg/mL. Sequences of shAhR was 5′-CCGGAGAGCTCTT TCCGGATAATAACTCGAGTTATTATCCGGAAAGAGCTCTTTTTTTG-3′.

### Intracisterna magna and intracerebral injection

Mice were anesthetized by intraperitoneal injection of a mixed solution of ketamine (100 mg/kg) and xylazine (10 mg/kg) in saline. For intracisterna magna injection, the skin of the neck was shaved and cleaned with 70% ethanol. A 50 μL Hamilton syringe was used to deliver a 10 μL bolus injection of viral suspension diluted at 10^12^ vg/mL or Alexa Fluor 555-conjugated ovalbumin (OVA, Thermo Fisher Scientific, USA, O34782) into the cisterna magna at a rate of 2.5 μL/min. After injecting, the syringe was left in place for at least 2 min to prevent backflow of CSF. For intracerebral injection, the mice were maintained in a stereotaxic frame. A hole was drilled in the localization: Bregma AP, −3.0 mm, ML, ±1.3 mm, DV, −4.7 mm [33]. 1 μL bolus injection of Alexa Fluor 555-conjugated OVA was infused using a 10 μL glass Hamilton syringe at a rate of 0.2 μL/min. The mice were allowed to recover from anesthesia on warmed pad (about 37 °C). Mice were sacrificed at 30 min after OVA intracisterna magna injection or 2 h after OVA intracerebral injection.

### Statistical analysis

Data were expressed as mean ± standard error of the mean (SEM). Statistical analysis was performed by Graphpad Prism v9. Statistical significance was determined: using Student’s *t* test (comparison of two groups), using Mann–Whitney tests (comparison of two groups, non-parametric data), one-way ANOVA or two-way ANOVA (comparison of three or more groups). When ANOVA showed significant differences, comparisons between means were tested by Bonferroni’s multiple comparisons test or Tukey’s multiple comparisons test. Values of *p* < 0.05 were considered statistically significant.

## Results

### EAE mice display reduced levels of SCFAs-producing bacteria and SCFAs

**Fig. 1:**
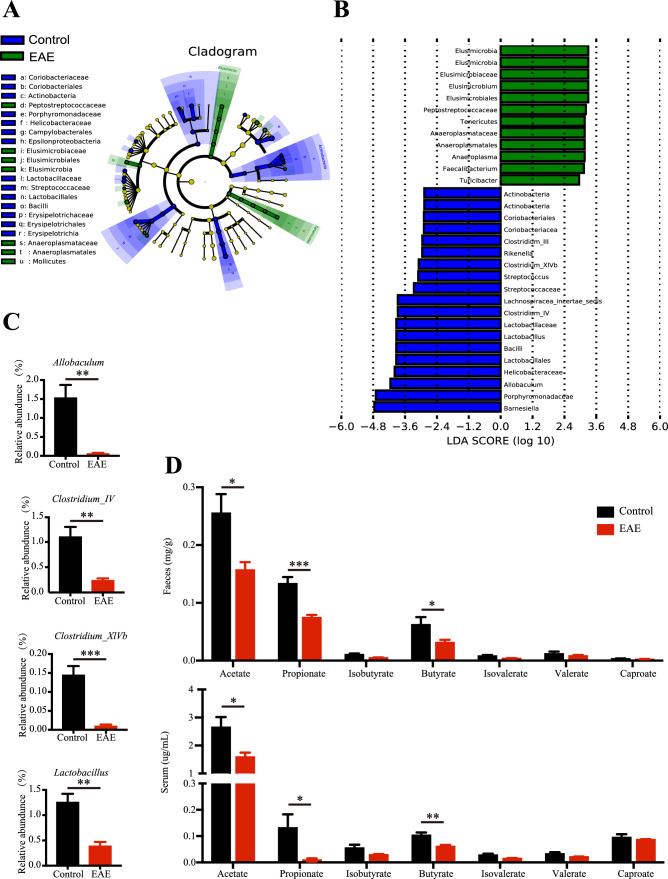
EAE mice display diminished presence of SCFAs-producing microbiota and decreased level of SCFAs. **A** Cladogram using LEfSe analysis indicated the phylogenetic distribution of gut microbiota. **B** LDA scores revealed a significant bacterial differences between control mice and EAE mice. **C** Relative abundances of SCFAs-producing microbiota were diminished in EAE mice. **D** SCFAs levels including acetate, propionate, isobutyrate, butyrate, isovalerate, valerate and caproate in both fecal and serum samples were quantified using LC–MS analysis. *n* = 6 in each group. Data were displayed as mean ± SEM. * *p* < 0.05, ** *p* < 0.01, *** *p* < 0.001. Statistical significance was determined using Student’s *t* test

It is widely accepted that SCFAs have a key role in the microbiota–gut–brain communication [1]. We analyzed the intestinal microbiota from EAE mice by 16S rRNA sequencing analysis. LEfSe and LDA score showed the differential distribution of microbiota between control-treated mice and EAE mice (Fig. 1A, B). We observed that the abundance of SCFAs-producing bacteria including *Allobaculum*, *Clostridium_IV*, *Clostridium_XlVb* and *Lactobacillus* genera were decreased in EAE mice compared to control-treated mice (Fig. 1C). By utilizing LC–MS analysis, we revealed a notable decrease in the concentrations of SCFAs, including acetate, propionate and butyrate, in both the feces and serum of EAE mice (Fig. 1D).

### SCFAs supplementation suppresses disease severity and astrocyte activation in EAE mice

**Fig. 2:**
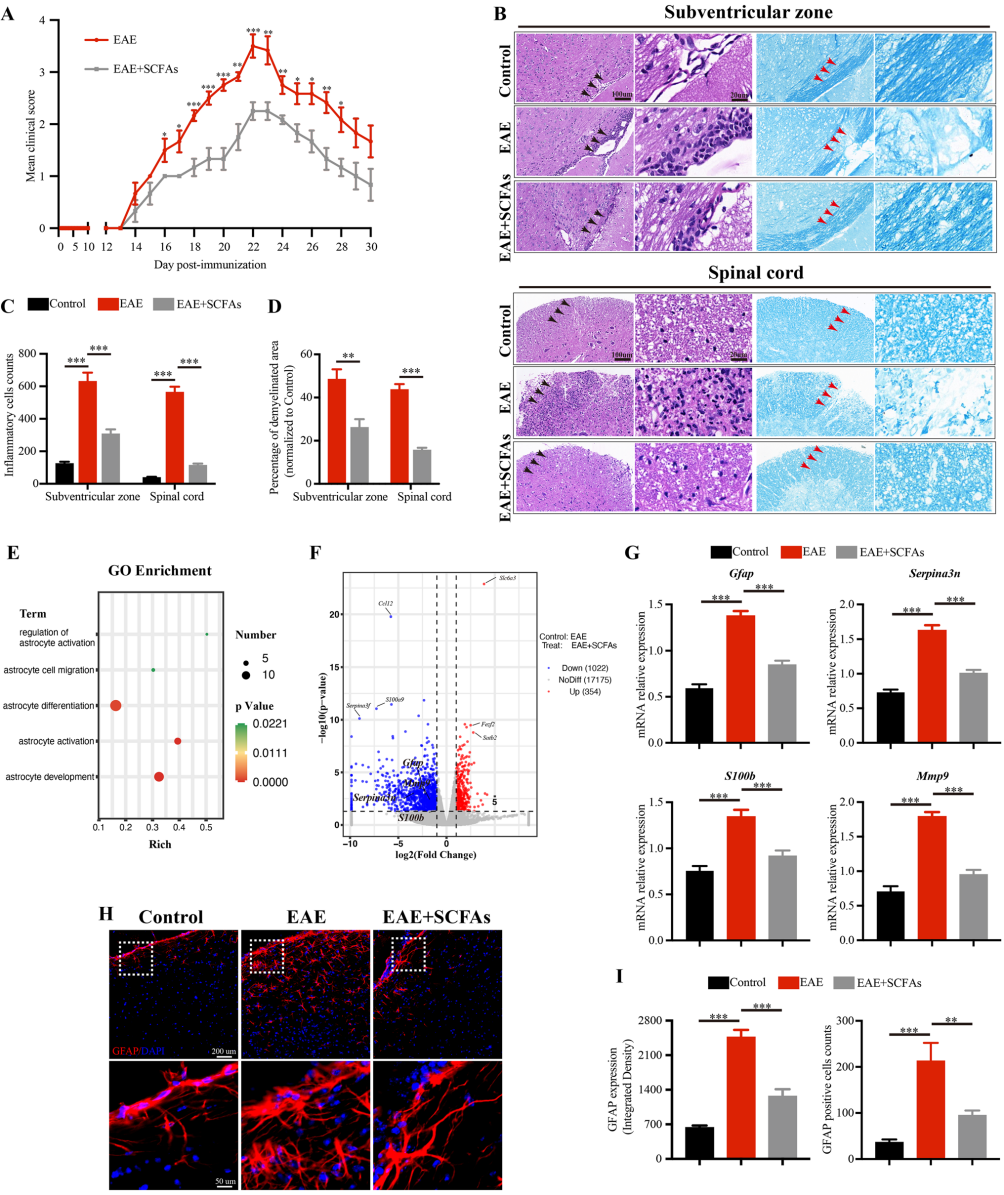
SCFAs supplementation suppresses disease severity and astrocytes activation in EAE mice. **A** Clinical scores of EAE mice and SCFAs-treated EAE mice were assessed daily and blindly by two researchers individually. *n* = 6 in each group. **B** On the 22nd day p.i. to initiate EAE, tissues of brains and lumbar spinal cords were isolated and were stained with HE and LFB, and the representative plots were shown, scale bars: 100 μm. Magnified images were displayed in the right column of the panels, scale bars: 20 μm. The black arrow pointed to the inflammatory cells. The red arrow pointed to the demyelinated area. **C**,**D** Quantification of the inflammatory cells counts and percentage of demyelinated area in different groups mice. *n* = 6 in each group. RNA-seq analysis were used to detected the relative expression of mRNA in brains between EAE mice and SCFAs-treated EAE mice. **E** GO enrichment analysis revealed 38 differentially expressed genes were linked to the biological process of astrocytes. **F** Volcano plots showed the expression profiles of the differentially expressed genes under SCFAs-treated and control-treated EAE mice. Blue dots and red dots represent down- and up-regulated differentially expressed genes, respectively (*p* value ≤ 0.05, fold change ≥ 2.0). *n* = 3 in each group. **G** RT-PCR was performed to detect the relative expression of mRNA of genes including *Gfap*, *S100b*, *Serpina3n* and *Mmp9* among Control mice, EAE mice and SCFAs-treated EAE mice. *n* = 6 in each group. **H**,**I** Representative immunofluorescent staining and statistical analysis of astrocytes activation in the subventricular zone, as indicated by GFAP (Red) fluorescence intensity analyzed with Image J software, and GFAP positive cells counts analyzed with HALO.® Highplex FL system, scale bars: 200 μm. Magnified images were displayed in the lower column of the panels, scale bars: 50 μm. *n* = 6 in each group. Data were displayed as mean ± SEM. * *p* < 0.05, ** *p* < 0.01, *** *p* < 0.001. Statistical significance was determined using Student’s *t* test (**A**, **D**), two-way ANOVA + Bonferroni’s multiple comparisons test (**C**), and one-way ANOVA + Bonferroni’s multiple comparisons test (**G**, **I**)

To further investigate the effects of SCFAs on EAE, a mixture of acetate, propionate, and butyrate (SCFAs) was added to the drinking water of EAE mice prior to disease induction. Control mice received drinking water that was salt and pH balanced. As shown in Fig. 2A, our observations revealed no significant variation in the timing of disease onset (spanning from the 13th to the 15th day p.i.) or in the disease severity during this period between EAE mice and SCFAs-treated EAE mice. Furthermore, we found that SCFAs supplementation led to a decrease in the clinical score of EAE mice from the 16th to the 28th day p.i. For the histopathology assessment, brains and lumbar spinal cords tissues were isolated on the 22nd day p.i. to initiate EAE. In line with the alleviation of clinical signs of EAE, our study revealed a reduction in the infiltration of inflammatory cells and an increase in the demyelinated area in the brains and lumbar spinal cords of EAE mice following SCFAs supplementation (Fig. 2B–D).

To elucidate the mechanisms by which SCFAs may reduce EAE severity, we analyzed mRNA expression in brains using RNA sequencing (RNA-seq). We detected 18,551 expressed genes and identified 1,376 transcripts that were differentially regulated in SCFAs-treated mice compared with EAE mice. GO enrichment analysis revealed that the differentially expressed genes were linked to the biological processes of astrocytes, including astrocyte activation (Fig. 2E, F). We further validated a significant downregulation of mRNA of genes following SCFAs supplementation using RT-PCR, including *Gfap*, *S100b*, *Serpina3n* and *Mmp9*, which are associated with astrocyte activation (Fig. 2G). Glial fibrillary acidic protein (GFAP) is an intermediate filament protein predominantly expressed in astrocytes and commonly used as a marker that represents the activation state of astrocytes [23]. In line with the results of RT-PCR, we observed an increased levels of GFAP expression and GFAP positive cell counts in EAE mice, which could be effectively diminished by SCFAs supplementation (Fig. 2H, I). Our findings indicate that the protective effect of SCFAs on EAE mice may be attributed to the suppression of astrocyte activation.

### SCFAs supplementation boosts tryptophan-derived AhR signaling in astrocytes of EAE mice

**Fig. 3:**
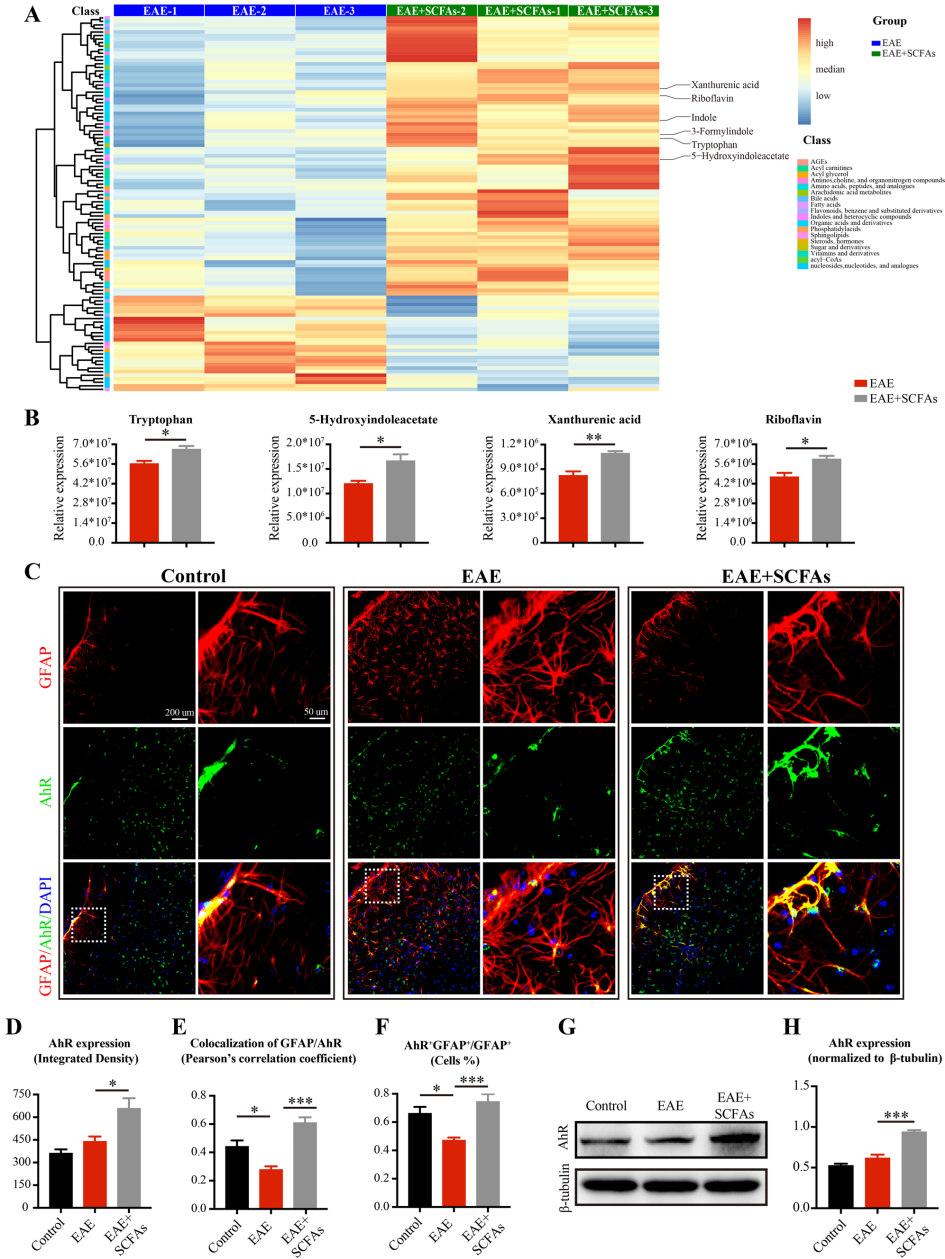
SCFAs supplementation boosts tryptophan-derived AhR signaling in astrocytes of EAE mice. **A** Differential metabolites in EAE mice induced by SCFAs supplementation were measured by large scale medical targeted metabolomics based on UHPLC-MS. **B** The statistical analysis of the relative expression of tryptophan, 5-hydroxyindoleacetate, xanthurenic acid, and riboflavin in EAE mice and SCFAs-treated EAE mice. *n* = 3 in each group. **C**–**F** Representative immunofluorescent staining and statistical analysis of the AhR expression within astrocytes in the subventricular zone, as indicated by AhR (green) fluorescence intensity analyzed with Image J software, colocalization of GFAP (red) and AhR (green) analyzed with Fiji software, and the proportion of AhR^+^GFAP^+^astrocytes analyzed with HALO.® Highplex FL system, scale bar: 200 µm. Magnified images were displayed in the right column of the panels, scale bars: 50 μm. *n* = 6 in each group. **G**,**H** Western blots and statistical analysis of the AhR protein expression in the brains of control mice, EAE mice, and SCFAs-treated EAE mice. *n* = 6 in each group. Data were displayed as mean ± SEM. * *p* < 0.05, ** *p* < 0.01, *** *p* < 0.001. Statistical significance was determined using Mann–Whitney tests (**B**), one-way ANOVA + Bonferroni’s multiple comparisons test (**D**, **E**, **H**) and one-way ANOVA + Tukey’s multiple comparisons test (**F**)

To elucidate the underlying mechanism of how SCFAs regulate astrocyte activation in EAE mice, we conducted large scale medical targeted metabolomics based on UHPLC-MS. Our findings revealed an elevation in the levels of Trp and its-derived AhR ligands, including 5-HIAA, xanthurenic acid, and riboflavin, following SCFAs supplementation in EAE mice (Fig. 3A, B). While indole and 3-formylindole showed a trend of increase in SCFAs-treated mice, the rise did not reach statistical significance. These results revealed a modification in the Trp-AhR signaling following SCFAs supplementation in EAE mice.

To further validate our findings, we assessed the expression of AhR in astrocytes by co-staining with GFAP and AhR in the subventricular zone of brain slices. We observed a reduction in the colocalization of GFAP and AhR, as well as a decrease in the proportion of AhR^+^GFAP^+^astrocytes within GFAP^+^astrocytes in EAE mice. Importantly, these alterations were reversed by SCFAs supplementation (Fig. 3C–F). Consistent with the immunofluorescent analysis, an elevated level of AhR protein was detected in the brain tissue of EAE mice following SCFAs supplementation (Fig. 3G, H). Our results conclusively demonstrate that SCFAs supplementation enhances Trp-AhR signaling in astrocytes of EAE mice.

### SCFAs supplementation suppression of astrocyte activation is AhR dependent

**Fig. 4:**
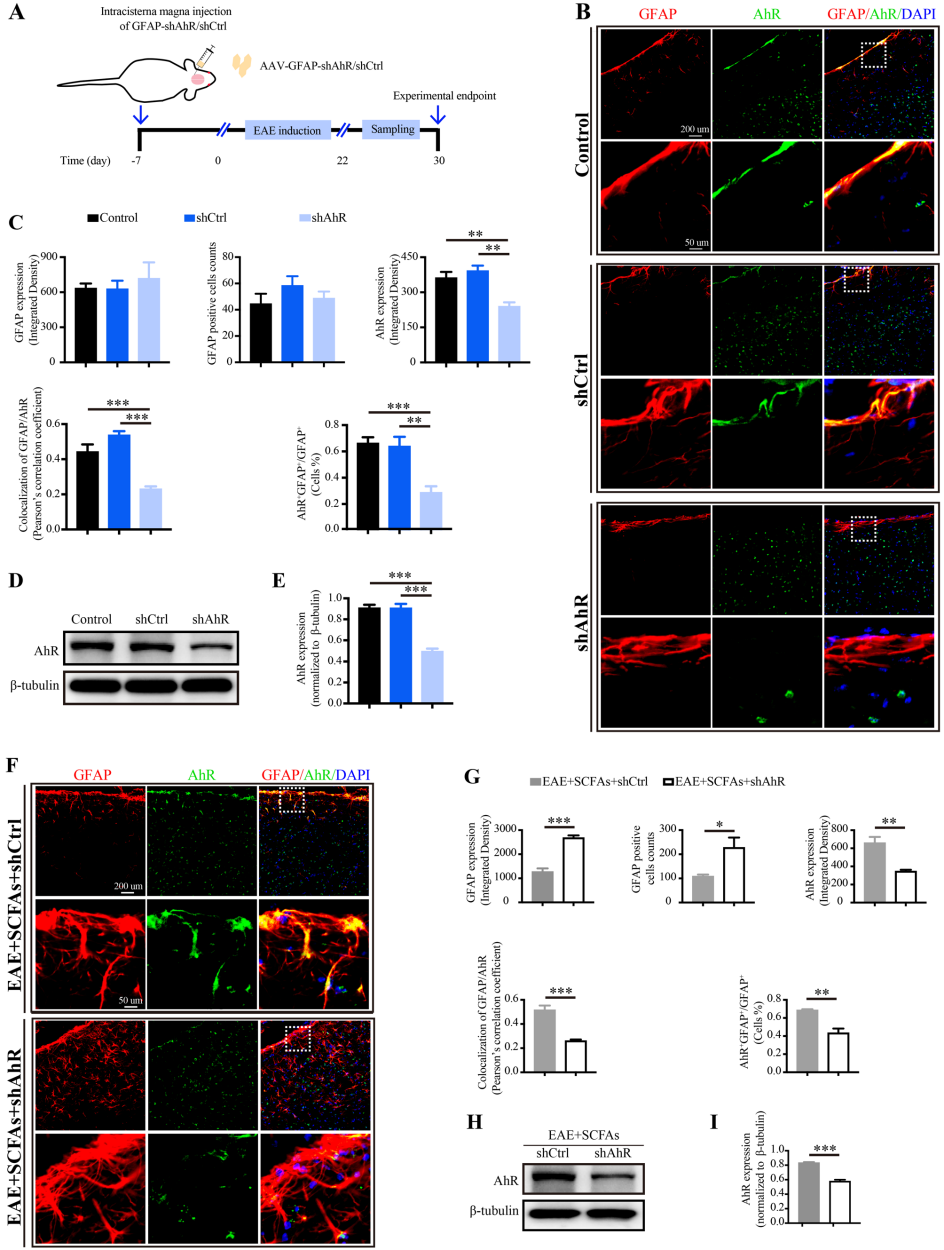
Intracisterna magna injection of AAV-GFAP-shAhR significantly reduces AhR expression in astrocytes. **A** This diagram illustrated the intracisterna magna injection of adeno-associated virus (AAV)-GFAP- shAhR/shCtrl and the experimental procedure. **B**,**C** Representative immunofluorescent staining plots and statistical analysis showed that intracisterna magna injection of GFAP-shAhR suppresses AhR (green) expression within astrocytes (GFAP, red) in the subventricular zone of control-treated mice. However, GFAP-shCtrl did not exhibit the same effect, scale bar: 200 µm. Magnified images were displayed in the lower column of the panels, scale bars: 50 μm. **D**,**E** Western blots and statistical analysis of the AhR protein expression in the brains of control-treated mice, GFAP-shCtrl mice and GFAP-shAhR mice. **F**,**G** Representative immunofluorescent staining plots and statistical analysis showed that GFAP-shAhR suppressed AhR (green) while increased GFAP (red) expression in astrocytes in SCFAs-treated EAE mice, scale bar: 200 µm. Magnified images were displayed in the lower column of the panels, scale bars: 50 μm. **H**,**I** Western blots and statistical analysis of the AhR protein expression in the brains of SCFAs-treated EAE mice with GFAP-shCtrl or GFAP-shAhR treatment. *n* = 6 in each group. Data were displayed as mean ± SEM. * *p* < 0.05, ** *p* < 0.01, *** *p* < 0.001. Statistical significance was determined using one-way ANOVA + Bonferroni’s multiple comparisons test (**C**, **E**) and Student’s *t* test (**G**, **I**)

In order to investigate the regulatory role of AhR expression in astrocyte activation and EAE disease, we utilized an adeno-associated viral (AAV) vector carrying a short hairpin RNA (shRNA) for mediating *Ahr* silencing under the control of the GFAP promoter (GFAP-shAhR). Mice received intracisterna magna injection of GFAP-shAhR or GFAP-shCtrl 1 week prior to EAE induction (Fig. 4A). Upon administration of GFAP-shAhR in control-treated mice, significant reductions in AhR expression, colocalization of GFAP and AhR, and the proportion of AhR^+^GFAP^+^astrocytes within GFAP^+^astrocytes were observed, while the GFAP expression and GFAP positive cell counts remained unaffected (Fig. 4B–E). Similarly, in SCFAs-treated EAE mice, the levels of AhR expression, colocalization of GFAP and AhR, and the proportion of AhR^+^GFAP^+^astrocytes within GFAP^+^astrocytes were reduced following GFAP-shAhR microinjection. Moreover, AhR downregulation via GFAP-shAhR also led to elevated GFAP expression and an increase in GFAP-positive cell counts in SCFAs-treated EAE mice (Fig. 4F–I). These findings indicate that AhR plays a critical role in mediating the suppressive effects of SCFAs supplementation on astrocyte activation.

### Amelioration of BBB-glymphatic dysfunction and EAE severity by SCFAs supplementation requires the expression of AhR in astrocytes

**Fig. 5:**
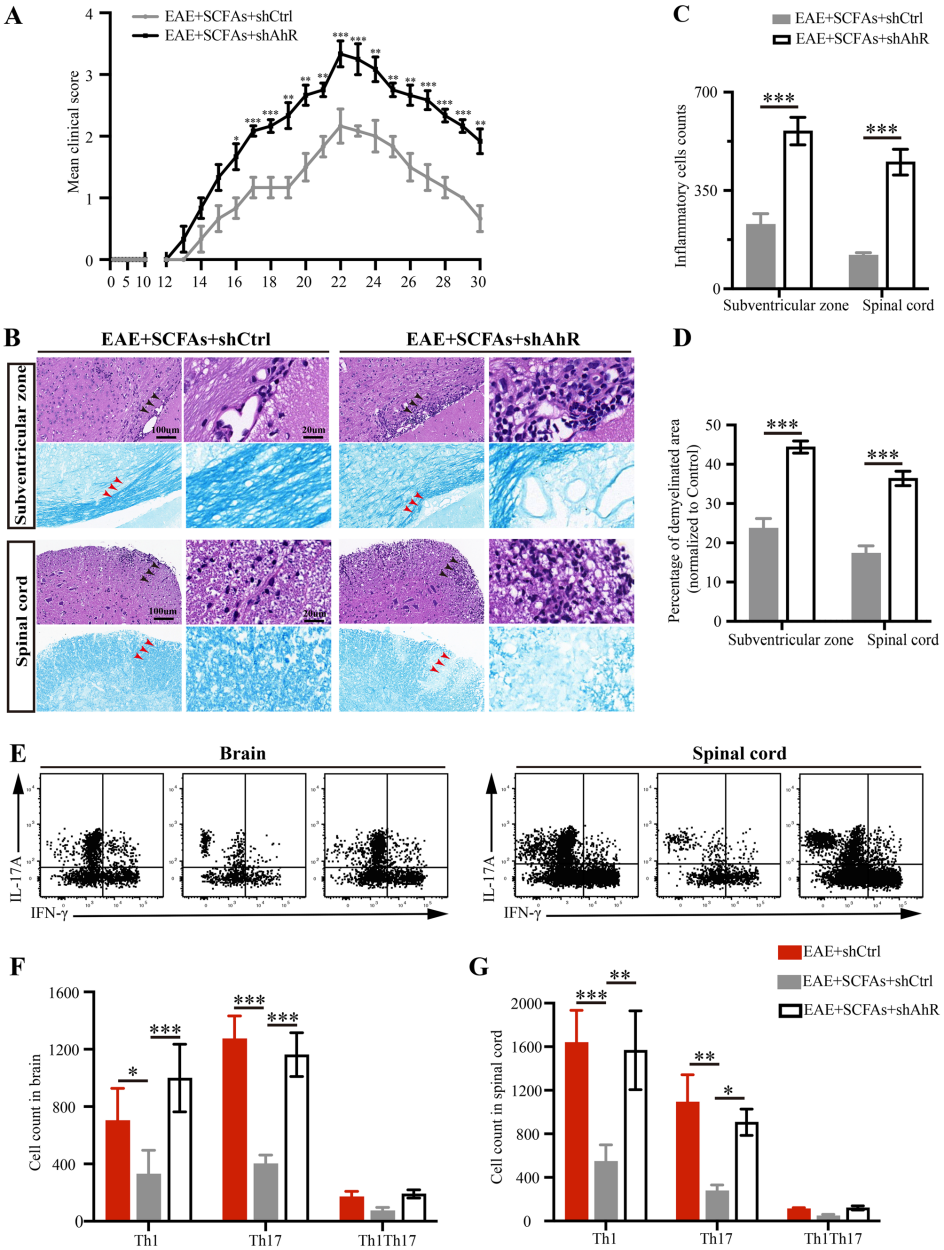
Amelioration of EAE disease by SCFAs supplementation requires the expression of AhR in astrocytes. **A** Daily clinical scores of SCFAs supplemental EAE mice treated with shCtrl or shAhR. **B**–**D** Representative plots and statistical analysis of HE and LFB staining of brain and lumbar spinal cords in SCFAs-treated EAE mice with GFAP-shCtrl or GFAP-shAhR treatment, scale bars: 100 μm. Magnified images were displayed in the right column of the panels, scale bars: 20 μm. The black arrow pointed to the inflammatory cells. The red arrow pointed to the demyelinated area. **E**–**G** Representative flow cytometry plots and statistical analysis showed the count of Th1 (CD4^+^IFN-γ^+^) cells, Th17 (CD4^+^IL-17A^+^) cells and Th1Th17 (CD4^+^IFN-γ^+^IL-17A.^+^) cells in different groups of mice. *n* = 6 in each group. Data were displayed as mean ± SEM. * *p* < 0.05, ** *p* < 0.01, *** *p* < 0.001. Statistical significance was determined using Student’s *t* test (**A**, **C**, **D**) and two-way ANOVA + Tukey’s multiple comparisons test (**F**, **G**)

We further found that SCFAs supplementation failed to ameliorate clinical signs and histopathology of EAE mice when AhR^+^astrocytes were reduced by GFAP-shAhR injection (Fig. 5A–D). Analysis of the T cell compartment revealed a reduction in the infiltration of Th1 (CD4^+^IFN-γ^+^) cells and Th17 (CD4^+^IL-17A^+^) cells in the brains and spinal cords of SCFAs-supplemented EAE mice compared to control-treated EAE mice. However, the downregulation of AhR expression in astrocytes in EAE mice abolished the suppressive effects of SCFAs on T cells infiltration (Fig. 5E–G). These results pinpoints the requirement of AhR expression in astrocytes for the SCFAs-mediated amelioration of EAE.

**Fig. 6:**
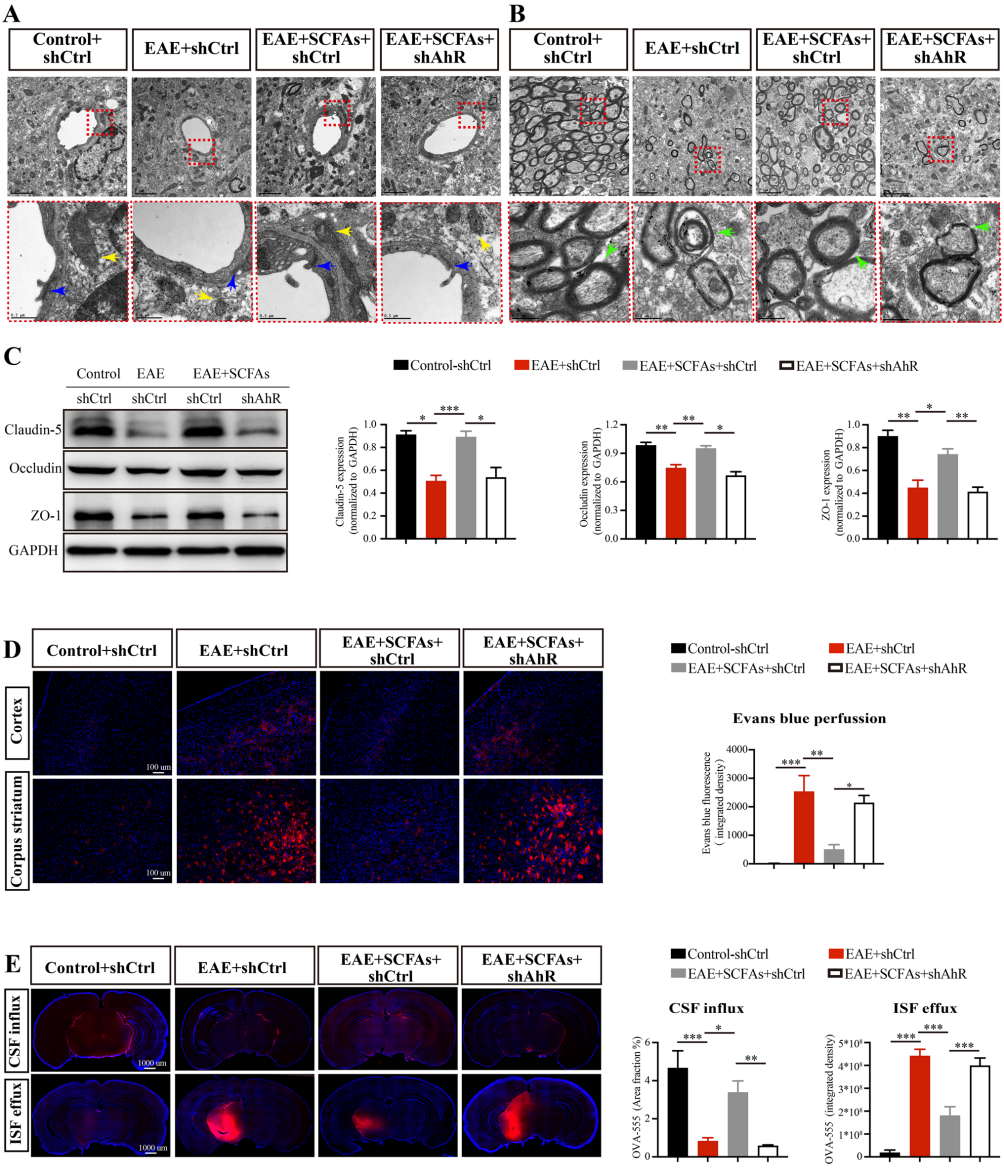
Amelioration of BBB-glymphatic dysfunction by SCFAs supplementation requires the expression of AhR in astrocytes. **A**,**B** Representative TEM plots of showed the ultrastructure of BBB and myelin sheath in different groups mice. The blue arrow pointed to the tight junction structure. The yellow arrow pointed to the mitochondria of astrocytes. The green arrow pointed to the myelin sheath structure. Scale bar: 0.5 µm. **C**,**D** Western blots and statistical analysis of the proteins expression of Claudin-5, Occludin and ZO-1 in the brains of different groups of mice. **E**,**F** Evans blue perfusion in the cortex and corpus striatum were measured to assess the BBB permeability. Scale bar: 100 µm. **G**,**H** Intracisterna magna and intracerebral injection of AF555-OVA were used to assess CSF influx and ISF efflux of glymphatic system function. Scale bar: 1000 µm. *n* = 6 in each group. Data were displayed as mean ± SEM. * *p* < 0.05, ** *p* < 0.01, *** *p* < 0.001. Statistical significance was determined using one-way ANOVA + Bonferroni’s multiple comparisons test (**D**, **F**) and one-way ANOVA + Tukey’s multiple comparisons test (**H**)

Given that astrocytes participate in the formation and function of the BBB-glymphatic system, which jointly maintain immune homeostasis within the CNS [12, 13], we next investigated the role of AhR^+^astrocytes in BBB-glymphatic function in SCFAs supplemental EAE mice. TEM ultrastructural observations of the BBB revealed an indistinct appearance of tight junction structure and a mitochondrial disruption in astrocytes as indicated by the swollen mitochondria with disorganized or absent cristae in EAE mice (Fig. 6A). In addition, a myelin sheath loss was observed in EAE mice (Fig. 6B). Importantly, SCFAs supplementation induced a prominently visible tight junction structure, ameliorated mitochondrial disruption in astrocytes, and reduced myelin sheath loss. However, these beneficial effects were hindered by the knockdown of AhR in astrocytes (Fig. 6A, B). Subsequently, we examined the expression of the key proteins that involved in restricting the BBB permeability and found that supplementation with SCFAs increased the expression of Claudin-5, Occludin, and ZO-1 (Fig. 6C, D). In line with these protein expression changes, we found that supplementation with SCFAs significantly reduced the infiltration of Evans blue dye in the cortex and corpus striatum of EAE mice (Fig. 6E, F). Furthermore, our experiments using intracisterna magna and intracerebral injection of AF555-OVA demonstrated that SCFAs treatment significantly enhanced CSF influx and ISF efflux of molecular tracers within the glymphatic system in EAE mice. However, this enhancement was hindered when the expression of AhR in astrocytes was downregulated (Fig. 6G, H). Collectively, our findings reveal that SCFAs supplementation ameliorates BBB-glymphatic dysfunction and alleviates EAE severity, an effect that relies on the expression of AhR in astrocytes.

### SCFAs supplementation suppresses loss of AQP4 polarity depends upon the expression of AhR in astrocytes

**Fig. 7:**
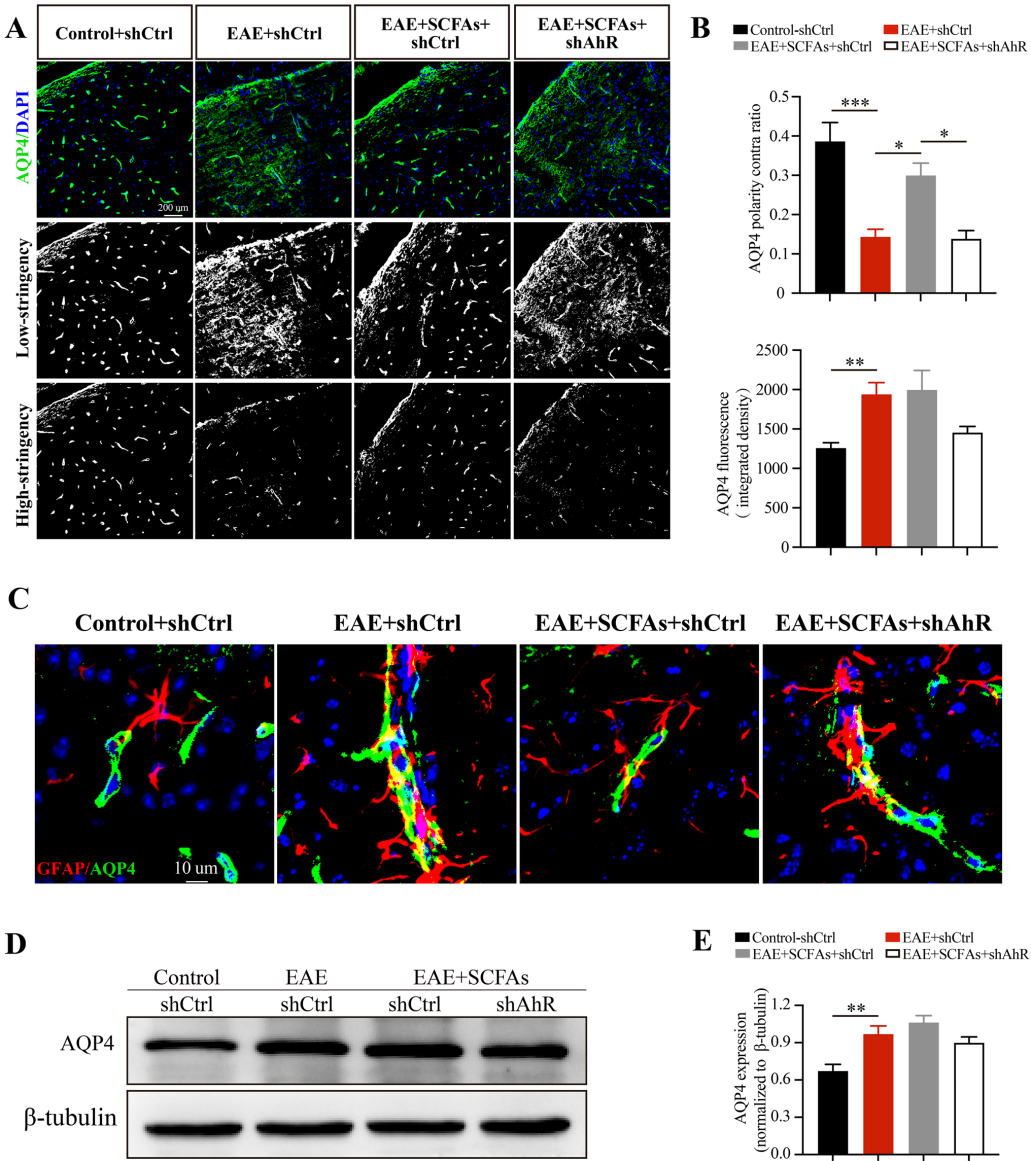
SCFAs supplementation suppresses loss of AQP4 polarity depends upon the expression of AhR in astrocytes. **A**,**B** Immunofluorescent staining and statistical analysis of AQP4 (green) in the subventricular zone of control mice, EAE mice and SCFAs-treated EAE mice with GFAP-shCtrl or GFAP-shAhR treatment. The low-stringency threshold was applied to define the overall area of AQP4 immunoreactivity, while the high-stringency threshold specifically identified the area of intense AQP4 immunoreactivity typically localized to perivascular end feet. AQP4 polarity contra ratio was calculated by the ratio of the high-stringency area to the low-stringency area. Scale bar: 200 µm. **C** Representative images of AQP4 polarity indicated by co-stained with GFAP (red) and AQP4 (green) in the astrocytes of control mice, EAE mice and SCFAs-treated EAE mice with GFAP-shCtrl or GFAP-shAhR treatment, Scale bar: 10 µm. **D**,**E** Western blots and statistical analysis of the proteins expression of AQP4 in the brains of different groups mice. *n* = 6 in each group. Data were displayed as mean ± SEM. * *p* < 0.05, ** *p* < 0.01, *** *p* < 0.001. Statistical significance was determined using one-way ANOVA + Bonferroni’s multiple comparisons test

AQP4 polarity, characterized by its high localization in the endfeet of astrocytes, plays an essential role in the mediation of astrocyte activation and BBB-glymphatic function [23–25]. Our RNA-seq and RT-PCR results reveal that SCFAs supplementation reduced the mRNA expression of matrix metalloproteinase 9 (Mmp9) in EAE mice, a factor that plays a key role in the loss of AQP4 polarity (Fig. 2E, F) [34]. To quantify AQP4 polarity, we applied uniform thresholds to images at two different levels, a high-stringency threshold specifically identifying the intense AQP4 immunoreactivity typically localized to perivascular end feet, and a low-stringency threshold defining the overall area of AQP4 immunoreactivity [35]. The ratio of the high-stringency area to the low-stringency area was calculated, resulting in an arbitrary value that represents the “AQP4 polarity” (Fig. 7A, B). In control and SCFAs-treated EAE mice, AQP4 expression is normally polarized insofar as it is expressed within the astrocytic end feet and not in the astrocytic somata. While in EAE mice and SCFAs + shAhR-treated EAE mice, the immunofluorescence of AQP4 was predominantly observed within the astrocytic somata rather than in the astrocytic end feet (Fig. 7C). Notably, the protein expression of AQP4 remains unaffected by SCFAs supplementation or shAhR administration in EAE mice (Fig. 7A, B, D, E). Based on these findings, we propose the hypothesis that AhR-mediated AQP4 polarity may be involved in the mechanism of SCFAs suppressing astrocyte activation.

## Discussion

Gut microbiota-derived signals have been demonstrated to play key role in the pathophysiological processes of MS [36]. In this study, we reported that SCFAs are reduced potentially as a consequence of a decreased abundance of SCFAs-producing microbiome in EAE. We also reported that SCFAs supplementation activates AhR in astrocytes by increasing the availability of Trp-derived AhR’s ligands, which subsequently suppresses loss of AQP4 polarity and astrocyte activation. These resulted in the amelioration of BBB-glymphatic dysfunction and EAE severity.

We found a significant reduction in the abundance of *Allobaculum*, *Clostridium_IV*, *Clostridium_XlVb*, and *Lactobacillus* genera in EAE mice compared with control mice. These bacterial genera are widely recognized as SCFAs-producing bacteria [37–39]. Corresponding to the altered gut microbiome, the concentrations of acetate, propionate, and butyrate in the feces and serum of EAE mice were also found to be decreased. Moreover, these reductions of SCFAs was also observed in Chinese cohort of patients with MS, and it was negatively correlated to the frequency of regulatory T cells [5]. Another research published in *Science* confirmed the reductions in these components in germ-free mice, and administered a mixture of SCFAs to these mice revealed its effect on colonic regulatory T cells [28]. Based on these findings, we supplemented the mixture of SCFAs to EAE mice to determine whether alterations in SCFAs levels were associated with the progression of the disease. We found that SCFAs supplementation ameliorated the clinical score and pathological changes in EAE. Furthermore, our findings are also supported by preclinical evidences demonstrating a decreased SCFAs concentrations in the patients with stroke, traumatic brain injury as well as Parkinson disease, and SCFAs supplementation alleviated the disease severity via the microbiota–gut–brain communication [5–7].

We have previously reported that *Clostridium butyricum* intervention effectively reduced Th17 cells response by suppressing the activity of p38 mitogen-activated kinase and c-Jun N-terminal kinase signaling in EAE mice [9]. Study has found that SCFAs induced IL-10 production in lymphocytes by reprogramming their metabolic activity towards elevated glucose oxidation in EAE mice [40]. Additionally, SCFAs have emerged as powerful regulators of mitochondrial energy metabolism in the pathogenic process of hypoperfusion-induced colonic dysfunction [41]. Similarly, we also observed an improvement in mitochondrial ultrastructure in EAE mice following SCFAs supplementation. However, the precise mechanism underlying how SCFAs regulate CNS cells in MS remains unclear. Recently, it has been reported that SCFAs restricted oxidative stress via the reduction of reactive oxygen species and reactive nitrogen species in microglia in Parkinson’s disease mice. Additionally, SCFAs facilitated the differentiation of immature oligodendrocytes in mice with cuprizone-induced demyelination [42, 43]. Here, we identified a previously unrecognized role of SCFAs in astrocyte activation in EAE. Our results shown that SCFAs supplementation significantly decreased the expression of astrocytes inflammatory phenotype-related genes including *Gfap*, *S100b*, *Serpina3n* and *Mmp9* [44, 45]. Concurrently, we observed a suppression of astrocyte activation. Given the established role of activated astrocytes as key components in the immunopathology of MS [46], we postulate that SCFAs supplementation likely reduces the severity of EAE through the suppression of astrocyte activation. This finding suggests a potential therapeutic role for SCFAs in modulating astrocyte function in MS and related neuroinflammatory disorders.

The AhR, which is ubiquitously expressed in astrocytes, functions as an environmental sensor capable of integrating microbiota-derived signals to regulate complex transcriptional processes within cells [17, 20]. The production of endogenous AhR ligands is influenced by the intestinal microbiota [47, 48]. Notably, supplementation with SCFA butyrate has been shown to enhance the availability of AhR ligands, relying on a fully functional endogenous microbiota [20]. Additionally, the *Allobaculum*, *Clostridium_IV*, *Clostridium_XlVb*, and *Lactobacillus* genera increased by SCFAs supplementation in our study have been demonstrated to contain a tryptophanase enzyme, which can metabolize Trp into various AhR ligands [49–51]. Furthermore, this fact was verified by the increased levels of Trp-derived AhR ligands, including 5-HIAA, xanthurenic acid, and riboflavin, following SCFAs supplementation. Given the available evidence, we speculate that SCFAs might potentially promote the Trp-AhR axis by creating a favorable environment for the microbiota possessing the tryptophanase enzyme. The AhR ligands/AhR pathway in astrocytes plays a pivotal role in the biological processes of cells. Excessive accumulation of indoxyl-3-sulfate and indoxyl sulfate in chronic kidney disease, and elevated levels of kynurenine in acute ischemic stroke, have been reported to promote astrocyte activation via AhR activation [52–54]. In contrast, our study found that SCFAs supplementation induced AhR signaling in astrocytes while concurrently suppressing cell activation. Moreover, the suppressive effect of SCFAs on astrocyte activation was abrogated in GFAP-shAhR-treated mice. This similar effect has also been observed in laquinimod and curcumin, demonstrating concurrent AhR activation and astrocyte activation inhibition [21, 55]. Thus, we considered the multifaceted roles of AhR in astrocyte activation might be attributed to disease model-specific and ligand-specific [56]. Here, we infer that SCFAs suppressed the astrocyte activation by increasing the availability of Trp-derived AhR in EAE mice, which subsequently activates AhR.

We conducted further investigations to elucidate the involvement of SCFAs-induced AhR signaling in astrocytes throughout the disease course. Our findings revealed that SCFAs supplementation led to a reduction in the infiltration of Th1 and Th17 cells in the brain and spinal cord, both of which are known to be major contributors to pathogenesis of MS [57, 58]. This reduction in T cells infiltration was further reflected by a relief in the severity of EAE and an amelioration of pathological alterations. Notably, we didn’t observe the protective effect of SCFAs in mice lacking AhR^+^astrocytes. These suggest that AhR signaling in astrocytes play a fundamental role in SCFAs suppressing the neuroinflammatory response in MS. During neuroinflammatory diseases, the BBB functions to prevent toxins and pathogens from permeating into the brain while to eliminate them into the bloodstream via transport proteins. Meanwhile, the glymphatic system clears solutes from the interstitial space by facilitating paravascular CSF-ISF exchange. Throughout the progression of MS, the BBB-glymphatic system collaborate to limit the infiltration of T cells into the CNS [14, 15]. The endfeet of astrocytes closely envelope cerebral microvessels, participating in the formation of the BBB-glymphatic system [12, 13]. It has been reported that suppression of astrocyte activation improved BBB-glymphatic dysfunction as well as alleviated neuronal damage in rats with cerebral ischemia and reperfusion injury [9]. Our study further found that SCFAs supplementation ameliorated BBB-glymphatic dysfunction relying on the expression of AhR in astrocytes, indicating that SCFAs-AhR signaling ameliorated BBB-glymphatic dysfunction via the suppression of astrocytes activation, ultimately leading to the alleviation of EAE severity.

The AQP4 polarity, characterized by its expression in the endfeet of astrocytes, is required for a normal rate of water exchange across the blood–brain interface. Following the discovery that AQP4 serves as a possible autoantigen in neuromyelitis optica, another research has confirmed the loss of AQP4 polarity in chronic-active MS lesions [59]. Analogously, our research also observed the loss of AQP4 polarity in EAE mice. Further, we determined that SCFAs supplementation alleviated the loss of AQP4 polarity in an AhR-dependent manner, which aligns with the observed changes in astrocyte activation and BBB-glymphatic function. The loss of AQP4 polarity is considered to lead to Ca^2+^ overload and inhibits PPAR-γ/mTOR-dependent autophagy in astrocytes, which subsequently activates astrocytes and impairs BBB-glymphatic function in sepsis-associated encephalopathy [60–62]. These results indicate that SCFAs-AhR signaling might suppress the activation of astrocyte and the impairment of BBB-glymphatic function in an AQP4 polarity dependent manner. The polarity of AQP4 is primarily regulated by Mmp9/β-dystroglycan (β-DG) pathway. Specifically, the structural integrity of β-DG is critical for the basement membrane-astrocyte endfeet contact and AQP4 polarization, while Mmp9 can mediate the cleavage of β-DG and thus contribute to the loss of AQP4 polarity [34]. Although previous study has demonstrated that AhR activation increased Mmp9 expression in gastric cancer cells [63], our study found that SCFAs supplementation promoted AhR activation and suppressed the expression of Mmp9 in EAE mice. One possible explanation for this discrepancy is that the interaction between AhR and Mmp9 may vary in the distinct pathological processes of diseases. Additionally, as a transcriptional factor, AhR has been demonstrated to form a heterodimer with the AhR nuclear translocator that interacts with the dioxin-responsive elements (DREs) of AQP4 promoter, leading to the upregulation of AQP4 expression [64]. Further research is required to validate the precise mechanism of how SCFAs-AhR signaling modulates AQP4 polarity.

In summary, our study demonstrates that SCFAs supplementation suppressed astrocytes activation by amplifying Trp-AhR-AQP4 signaling, which has a beneficial effect on the BBB-glymphatic function and disease severity of EAE. Our findings suggest that SCFAs can serve as a potent immunomodulatory supplement to MS treatments.

### Supplementary Information

Below is the link to the electronic supplementary material.

Supplementary file1 (PDF 140 KB)

Supplementary file2 (PDF 152 KB)

Supplementary file3 (DOCX 15209 KB)

## Data Availability

The datasets generated during this study are available from the corresponding author upon reasonable request.

## Abbreviations

CNS: Central nervous system
SCFAs: Short-chain fatty acids
MS: Multiple sclerosis
EAE: Experimental autoimmune encephalomyelitis
BBB: Blood–brain barrier
AhR: Aryl-hydrocarbon receptor
Trp: Tryptophan
5-HIAA: 5-Hydroxyindoleacetate
AQP4: Aquaporin-4
CSF: Cerebrospinal fluid
ISF: Interstitial fluid
CFA: Complete Freund’s adjuvant
PLS-DA: Partial least squares discrimination analysis
OTU: Operational taxonomic units
LDA: Linear discriminant analysis
p.i.: Post-induction
TEM: Transmission electron microscopy
GFAP: Glial fibrillary acidic protein
AAV: Adeno-associated viral
Mmp9: Matrix metalloproteinase 9
β-DG: β-Dystroglycan
DREs: Dioxin-responsive elements
