# Supplementary figures and images for: Short-chain fatty acids suppresses astrocyte activation by amplifying Trp-AhR-AQP4 signaling in experimental autoimmune encephalomyelitis mice
Source: Cell Mol Life Sci. 2024 Jul 8;81(1):293. doi: 10.1007/s00018-024-05332-x (PMC11335219; doi:10.1007/s00018-024-05332-x)

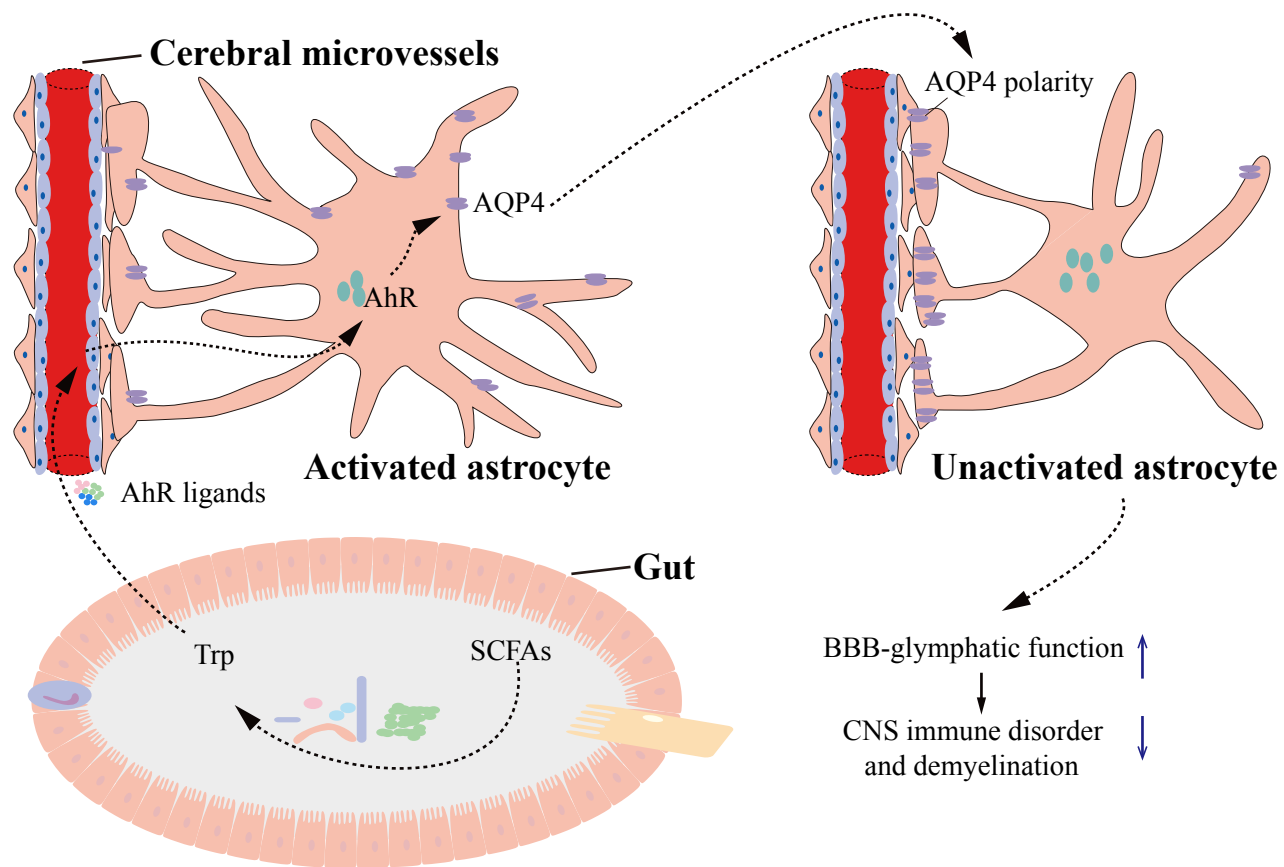

Supplement: Supplementary file 1 — Supplementary file1 (PDF 140 KB) [file 18_2024_5332_MOESM1_ESM.pdf]

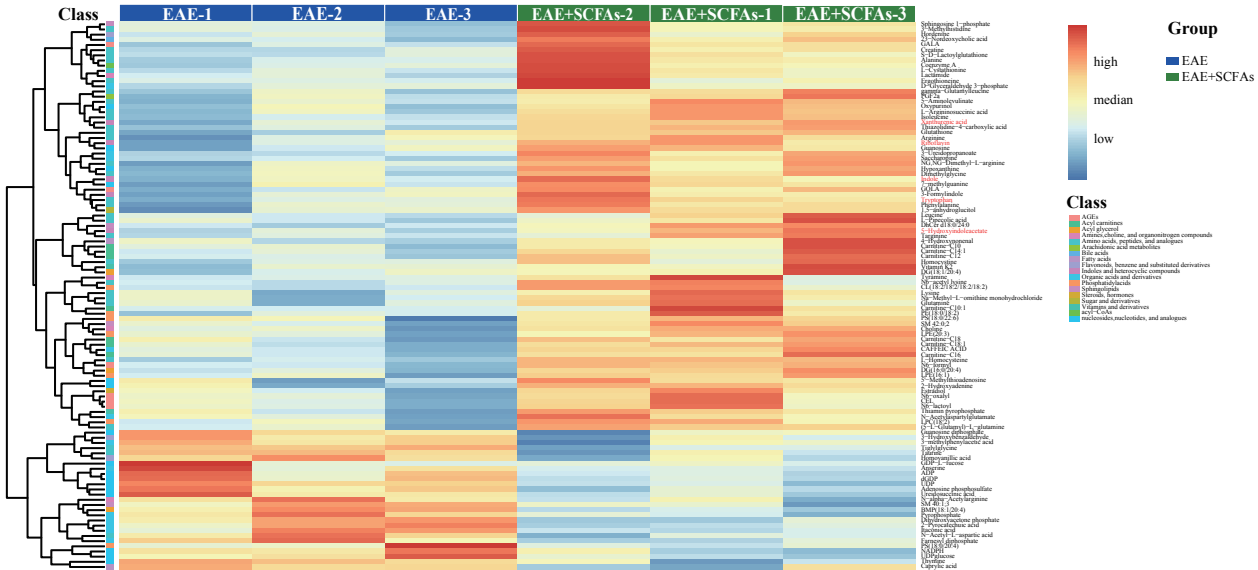

Supplement: Supplementary file 2 — Supplementary file2 (PDF 152 KB) [file 18_2024_5332_MOESM2_ESM.pdf]
