# Supplementary material for: Short-chain fatty acids suppresses astrocyte activation by amplifying Trp-AhR-AQP4 signaling in experimental autoimmune encephalomyelitis mice
Source: Cell Mol Life Sci. 2024 Jul 8;81(1):293. doi: 10.1007/s00018-024-05332-x (PMC11335219; doi:10.1007/s00018-024-05332-x)
Supplement: Supplementary file 3 — Supplementary file3 (DOCX 15209 KB) [file 18_2024_5332_MOESM3_ESM.docx]

**Supplementary Information**

**Short-chain fatty acids suppresses astrocyte activation by amplifying Trp-AhR-AQP4 signaling in experimental autoimmune encephalomyelitis mice**

**Supplementary Table 1.** Top 50 differentially expressed genes of the transcriptional profile of brain in EAE and SCFAs-treated EAE mice

| **Name of gene** | **log2FoldChange** | **p-value** |
| --- | --- | --- |
| Slc6a3 | 3.890504811 | 1.31767E-23 |
| Ccl12 | -5.777096452 | 1.64606E-20 |
| Tmem252 | -2.339082447 | 1.39667E-12 |
| S100a8 | -5.714100733 | 3.49421E-12 |
| S100a9 | -7.271460172 | 8.83744E-12 |
| Ly86 | -2.894721929 | 4.22758E-11 |
| Serpina3f | -9.0232692 | 7.65028E-11 |
| Acer2 | -1.926418175 | 2.67393E-10 |
| Myoc | 1.900078349 | 2.69928E-10 |
| Fezf2 | 2.498378544 | 3.28864E-10 |
| Vip | 2.07395369 | 5.08085E-10 |
| Ptpn7 | -4.259605036 | 1.32504E-09 |
| Satb2 | 2.774149517 | 1.62024E-09 |
| Ccl8 | -5.578466358 | 3.73053E-09 |
| Cxcr2 | -Inf | 3.9752E-09 |
| Bdnf | 1.412593198 | 4.15151E-09 |
| Itgam | -2.021248384 | 5.27836E-09 |
| Gm6614 | -5.557559949 | 5.60545E-09 |
| Xdh | -1.849631565 | 1.15936E-08 |
| Tnfrsf25 | 2.172180325 | 1.18969E-08 |
| Nfam1 | -2.02890144 | 1.53941E-08 |
| Glt8d2 | 1.837289815 | 2.35391E-08 |
| Inpp5d | -1.463476179 | 2.54746E-08 |
| Rasal3 | -2.608946263 | 2.72961E-08 |
| Slc30a3 | 1.655686772 | 2.86243E-08 |
| Fcgr3 | -2.557258651 | 2.93104E-08 |
| Col5a1 | 1.375746658 | 3.56128E-08 |
| Olfml3 | -1.402893219 | 4.44488E-08 |
| Rtn4r | 1.357660329 | 5.93379E-08 |
| Nr4a3 | 1.496332949 | 6.24681E-08 |
| Bin2 | -2.22235493 | 6.51476E-08 |
| Satb1 | 1.183469077 | 7.8945E-08 |
| Fcgr4 | -7.049648056 | 1.11682E-07 |
| Rtn4rl2 | 2.413197288 | 1.25464E-07 |
| Enpp2 | -1.067890021 | 1.55684E-07 |
| Entpd1 | -1.288684004 | 1.84819E-07 |
| Mical2 | 1.284709774 | 2.22462E-07 |
| Lingo1 | 1.6513083 | 2.44091E-07 |
| Slc38a5 | -2.000069752 | 2.4927E-07 |
| Tifab | -2.248958458 | 2.56878E-07 |
| Ikzf1 | -2.292833192 | 2.97879E-07 |
| Vxn | 2.103358367 | 3.06141E-07 |
| Tbr1 | 2.336913909 | 3.28631E-07 |
| Dkk3 | 1.570631611 | 3.35614E-07 |
| Mef2c | 1.968054922 | 3.78314E-07 |
| Cd14 | -3.241768515 | 4.16036E-07 |
| Pros1 | -1.240778166 | 4.70528E-07 |
| Epop | 1.762595803 | 4.76372E-07 |
| Tmem176b | -1.387243938 | 5.29951E-07 |
| Tnfsf8 | -6.195981885 | 5.5441E-07 |

**Supplementary Table 2.** Top 20 biological processes of the transcriptional profile of brain in EAE and SCFAs-treated EAE mice

| **Name of gene set** | **Up** | **Down** | **DEG** | **Total** | **Pvalue** |
| --- | --- | --- | --- | --- | --- |
| immune system process | 29 | 509 | 538 | 2640 | 7.077E-158 |
| immune response | 15 | 388 | 403 | 1554 | 1.1419E-150 |
| defense response | 24 | 370 | 394 | 1672 | 2.6907E-131 |
| regulation of immune system process | 18 | 328 | 346 | 1439 | 3.1966E-116 |
| response to external stimulus | 63 | 433 | 496 | 2917 | 1.9048E-109 |
| defense response to other organism | 8 | 284 | 292 | 1108 | 2.3128E-107 |
| response to external biotic stimulus | 12 | 331 | 343 | 1513 | 4.6695E-107 |
| response to other organism | 11 | 331 | 342 | 1509 | 1.1321E-106 |
| response to biotic stimulus | 12 | 333 | 345 | 1552 | 4.2298E-105 |
| positive regulation of immune system process | 14 | 248 | 262 | 958 | 1.35516E-99 |
| innate immune response | 7 | 236 | 243 | 829 | 4.19082E-99 |
| interspecies interaction between organisms | 12 | 343 | 355 | 1730 | 1.28072E-97 |
| immune effector process | 6 | 232 | 238 | 844 | 5.25963E-93 |
| regulation of immune response | 6 | 218 | 224 | 757 | 7.14489E-92 |
| regulation of response to external stimulus | 23 | 228 | 251 | 1032 | 4.26813E-83 |
| leukocyte activation | 20 | 216 | 236 | 952 | 1.2489E-79 |
| cell activation | 22 | 228 | 250 | 1074 | 2.0574E-78 |
| regulation of defense response | 14 | 183 | 197 | 688 | 1.38609E-77 |
| regulation of response to stimulus | 106 | 433 | 539 | 4066 | 2.55572E-76 |
| cytokine production | 8 | 197 | 205 | 771 | 1.66347E-74 |

**Supporting Figure Legends**

**Fig S1.** Schematic model of this study. SCFAs supplementation promote the catabolism of Trp in gut, generating high-abundance AhR ligands that recognize and activate AhR in astrocytes. The Trp-AhR signaling restores the polarity of AQP4, inhibits the activation of astrocytes, ameliorates BBB-glymphatic function and CNS immune disorder and demyelination in EAE.

**Fig S2.** SCFAs supplementation increase tryptophan-derived AhR ligands in EAE mice. Differential metabolites in EAE mice induced by SCFAs supplementation were measured by large scale medical targeted metabolomics based on UHPLC-MS.

**Supporting Uncropped Blots Images**

**Fig. 3E**


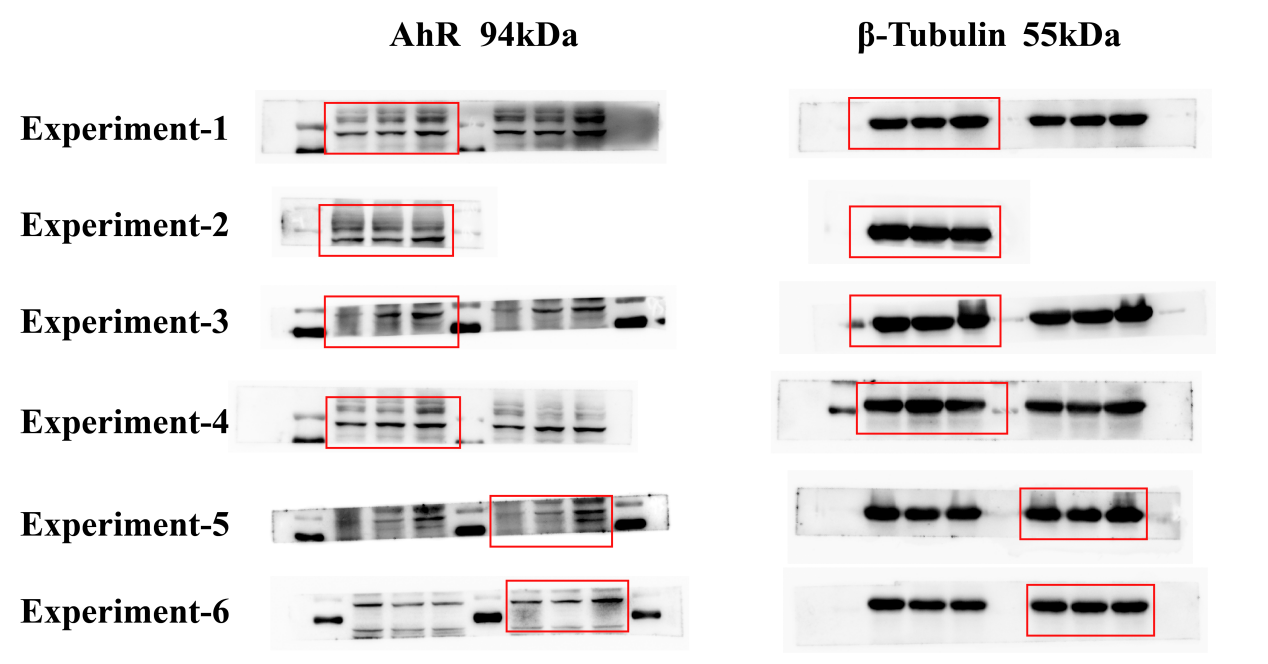


**Fig. 4D**

**
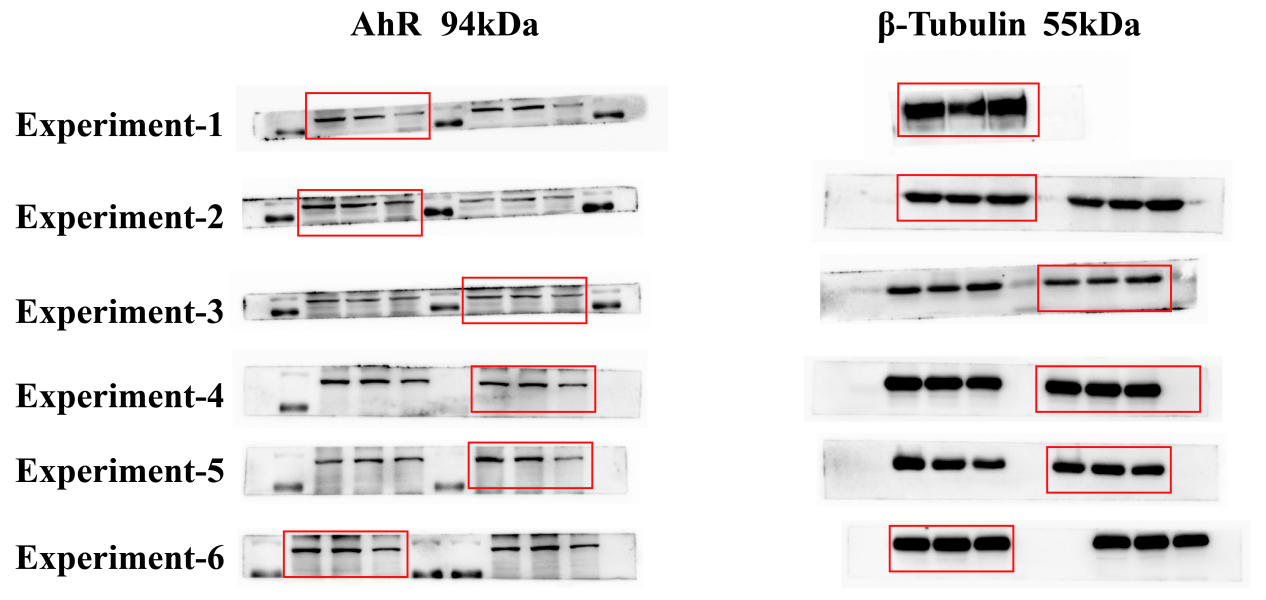
**

**Fig. 4H**


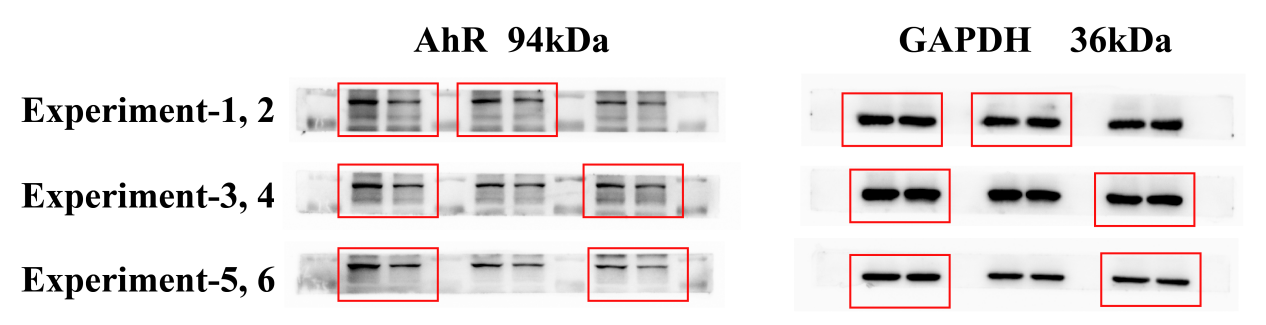


**Fig. 6C**


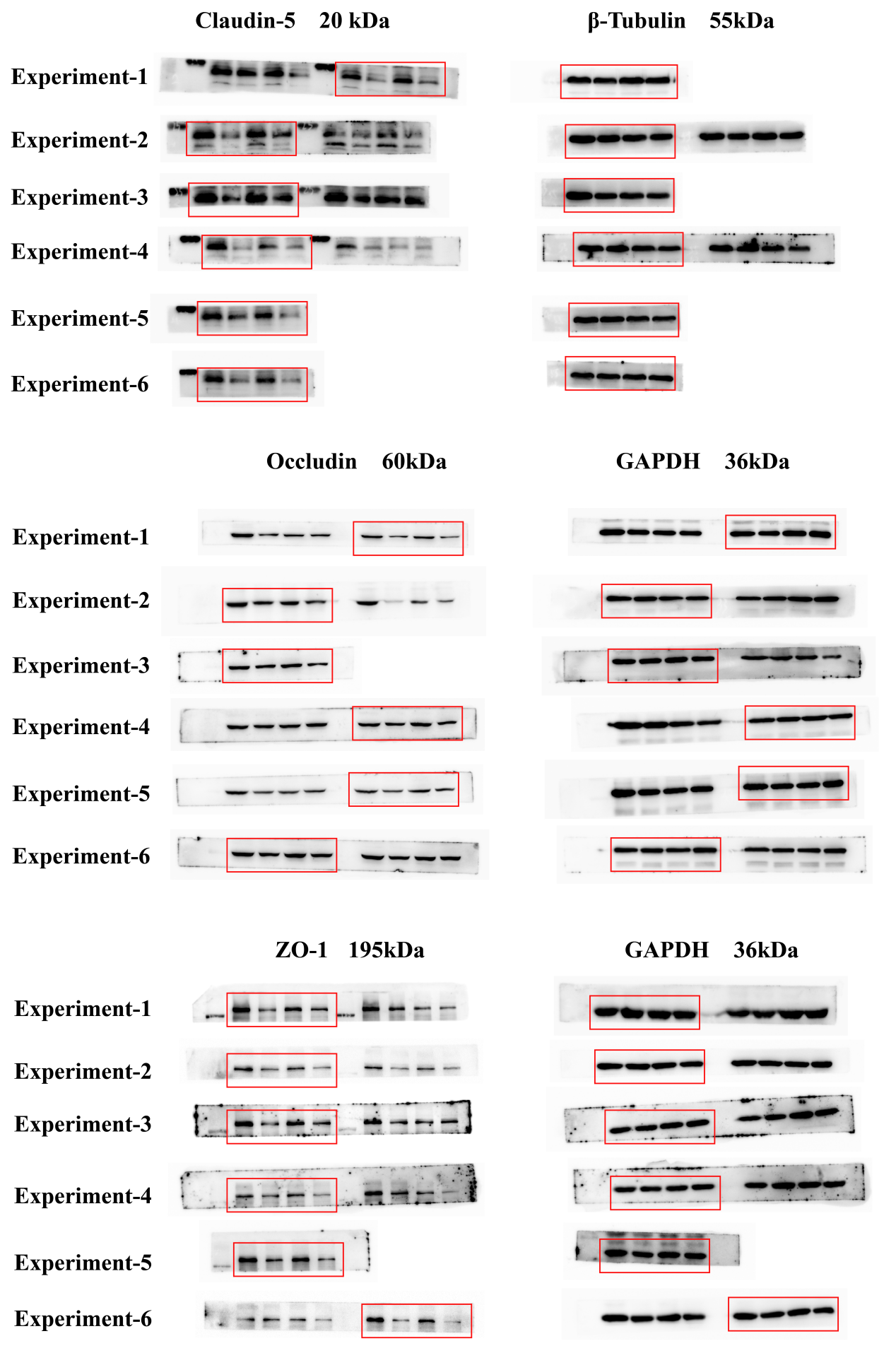


**Fig. 7D**

**
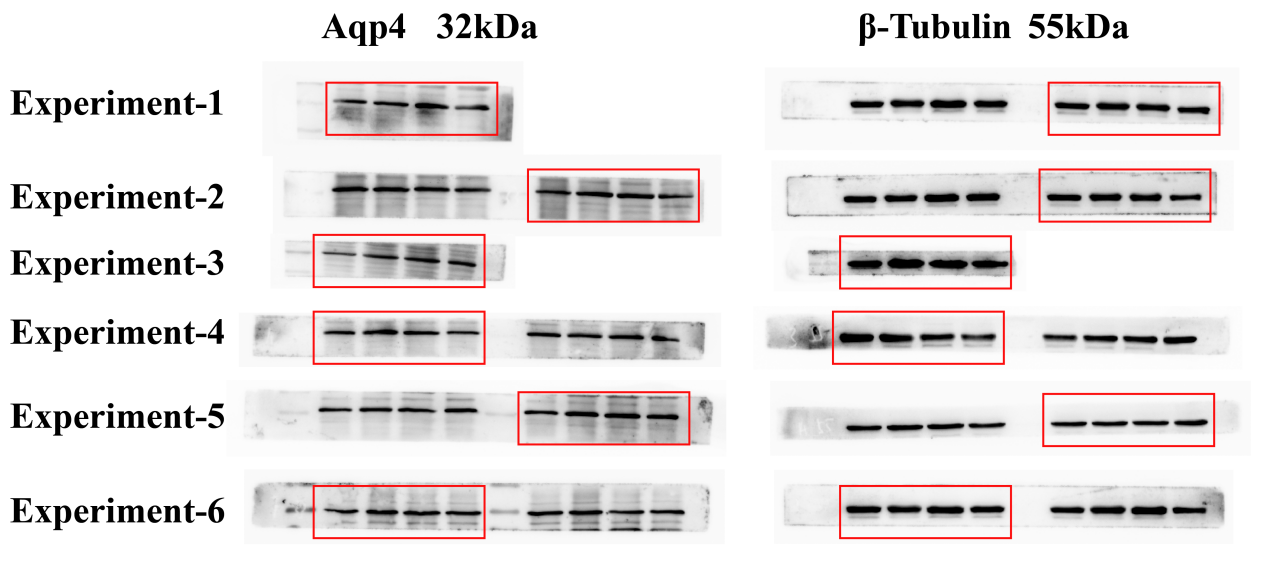
**
